# A randomised crossover trial of daridorexant for the treatment of chronic insomnia and nocturia

**DOI:** 10.1111/jsr.70002

**Published:** 2025-03-13

**Authors:** Katharina Lederer, Heike Benes, Alan Fine, Sylvia Shoffner, Sandro Bacchelli, David Castro Diaz, Jose Emilio Batista, Racheal Rowles, Tobias Di Marco, Michael Meinel

**Affiliations:** ^1^ Advanced Sleep Research GmbH Berlin Germany; ^2^ Somni Bene Institut für Medizinische Forschung und Schlafmedizin Schwerin GmbH Schwerin Germany; ^3^ Clinical Research Center of Florida Pompano Beach Florida USA; ^4^ Accellacare Research of Cary Cary North Carolina USA; ^5^ Encore Medical Research of Weston LLC Weston Florida USA; ^6^ Complejo Hospitalario Universitario de Canarias Tenerife Spain; ^7^ Uroclinica Barcelona Centro Médico Teknon Barcelona Spain; ^8^ Idorsia Pharmaceuticals Ltd Allschwil Switzerland

**Keywords:** daridorexant, dual orexin receptor antagonist, insomnia, nocturia, total sleep time, voids

## Abstract

This double‐blind, placebo‐controlled, two‐way crossover trial evaluated the efficacy and safety of daridorexant in patients with chronic insomnia and comorbid nocturia. In total, 60 patients aged ≥55 years with insomnia complaints for ≥3 months, Insomnia Severity Index (ISI) ≥13 and ≥3 voids/night for ≥1 month were randomised (1:1) to daridorexant 50 mg/placebo for 4 weeks followed by crossover after a 14–21‐day washout period. The primary endpoint was change from baseline to Week (W) 4 in self‐reported total sleep time (sTST). Other endpoints included change in ISI score, sleep depth and quality (visual analogue scale scores), nocturnal voids (mean number, time to first) and daytime functioning (Insomnia Daytime Symptoms and Impacts Questionnaire score [IDSIQ]). At W4, daridorexant significantly increased sTST versus placebo (least‐squares mean difference [LSMD] 20.9 min, 95% confidence interval [CI] 8.0–33.7; *p* = 0.002); significant improvements were also seen at W1–3. Compared with placebo, daridorexant significantly decreased (*p* < 0.001) ISI at both timepoints, W2 (LSMD −3.7, 95% CI −5.1 to −2.3) and W4 (LSMD −3.3, 95% CI −4.7 to −1.8) and significantly improved (*p* < 0.05) sleep depth (W1, 2, 3, 4), sleep quality (W1, 2, 3) and IDSIQ total score (W1, 3). Daridorexant versus placebo reduced the number of voids (LSMD [95% CI]: W1–0.6 [−0.9 to −0.3], *p* < 0.001; W4–0.3 [−0.7 to +0.1], *p* = 0.090) and increased median time to first void (difference to placebo, W1: +31 min, *p* = 0.0027; W4: +23 min, *p* = 0.2026). No adverse events of special interest (falls/urinary incontinence) were reported during daridorexant treatment. In conclusion, in patients with chronic insomnia and nocturia, daridorexant improves both conditions with a favourable safety profile.

## 1 INTRODUCTION

Chronic insomnia is defined as a subjective complaint of trouble initiating or maintaining sleep for ≥3 nights/week over a period ≥3 months combined with a significant negative impact on daytime functioning (American Academy of Sleep Medicine, 2014). Insomnia is one of the most common sleep conditions in Western countries, with approximately one‐third of adults experiencing difficulty sleeping and up to 10% fulfilling the diagnostic criteria for chronic insomnia disorder (Baglioni et al., 2020; LeBlanc et al., 2009; Morin & Jarrin, 2022; Riemann et al., 2023), which is associated with a high burden for the patient and an increased risk of cardiovascular, neurological and psychiatric diseases if left untreated (Gottesman et al., 2024; Javaheri & Redline, 2017; Palagini et al., 2022). Insomnia often coexists with nocturia, defined as the need to wake up at night to void, with each void preceded and followed by sleep/intention to sleep (Hashim et al., 2019; van Kerrebroeck et al., 2002). Both conditions occur more frequently with ageing and are associated with impairments in quality of life and daytime functioning (Ancoli‐Israel et al., 2011; Bliwise et al., 2019; Kyle et al., 2010), and increased risk of accident and fall (Grandner et al., 2023; Pesonen et al., 2020). While evidence shows nocturia to be a major cause of disturbed sleep (Ancoli‐Israel et al., 2011), the relationship is likely bidirectional (Bliwise et al., 2019).

Pharmaceutical therapies commonly used to manage nocturia (anticholinergics, alpha adrenergic blockers and antidiuretic hormone analogues) have shown limited efficacy at treating nocturia symptoms, probably due to its multifactorial causes (Paganini et al., 2015; Park & Kim, 2013). Given the high prevalence of sleep disruptions in patients with nocturia, studies have examined the effects of hypnotics in improving sleep and/or nocturia in these patients (Burke et al., 2024; Drake et al., 2018; Mukouyama et al., 2013). However, results have often been inconclusive or studies small and uncontrolled. Moreover, the risk of falls and accidents frequently associated with hypnotics (Miwa et al., 2011; Song & Ku, 2007) may be of particular concern in an older population that has to get up during the night to void (Nakagawa et al., 2010) underscoring the need for novel therapeutic options that are safe and effective for these patients.

Dual orexin receptor antagonists (DORAs), which allow sleep to occur by reducing the wake drive without causing broad sedation of the central nervous system, have, in the last decade, emerged as a new class of drug for the treatment of insomnia (Mogavero et al., 2023). Robust clinical studies assessing the efficacy and safety of DORAs in patients with insomnia and nocturia are nonetheless lacking. Suvorexant, the first approved DORA, has been studied in patients with both conditions but in a small, uncontrolled clinical study, making results difficult to interpret (Suetomi et al., 2019). In addition, the benefits of suvorexant on nocturia enuresis are limited to a single case study (Matsumoto, 2021). Similarly, in a recent study on lemborexant, another DORA, conducted in Japan, improvements in sleep and reductions in nocturia episodes were observed (Togo et al., 2024). However, the lack of blinding, randomisation, and a placebo arm limits the interpretation of the treatment effect and further emphasises the need for well‐controlled clinical trials.

Daridorexant is the most recent DORA developed and approved for the treatment of chronic insomnia (Markham, 2022). In patients with chronic insomnia, daridorexant 50 mg reduces time to sleep onset and wakefulness after sleep onset, improves total sleep time (TST) and daytime functioning without excessive morning residual effects and reduces the severity of insomnia as compared to placebo (Mignot et al., 2022).

This present clinical trial examined the efficacy and safety of daridorexant 50 mg in patients with chronic insomnia and nocturia. The primary objective was to evaluate the efficacy of daridorexant on self‐reported TST (sTST). The benefits of daridorexant on nocturia symptoms, daytime functioning and quality of life were also assessed.

## 2 METHODS

### 2.1 Trial design

This was a multi‐centre, double‐blind, randomised, placebo‐controlled, two‐way crossover trial conducted in 16 sites (clinical research units, hospitals, private offices) in Germany, Spain, and the United States (ClinicalTrials.gov identifier: NCT05597020). The crossover design was chosen for this study as it removes the inter‐subject variability from the comparison between treatment groups and reduces the effect of covariates. In addition, the crossover design allows for a smaller sample size compared to a parallel group design, leading to a higher power and a higher level of accuracy of the estimates. For these reasons, the study design was deemed appropriate in this chronic, long‐term condition with short lasting benefit, but no cure expected, from investigational treatment.

The trial was conducted in accordance with the International Conference on Harmonization guidelines for Good Clinical Practice and principles of the Declaration of Helsinki and local regulations. The protocol was approved by institutional review boards or independent ethics committees and all patients provided written informed consent. Trial methods and results are reported following the Consolidated Standards of Reporting Trials (CONSORT) guideline for randomised controlled crossover trials (Dwan et al., 2019).

### 2.2 Participants

Inclusion criteria included age ≥55 years, insomnia complaints for ≥3 months prior to screening, an Insomnia Severity Index (ISI) (Morin et al., 2011) score of ≥13 during screening, and waking to void during the main sleep period, with each void preceded and followed by sleep/intention to sleep (Hashim et al., 2019). Patients had to self‐report on average ≥3 nocturnal voiding episodes/night for ≥1 month prior to screening and report ≥2.6 voiding episodes/night over 3 consecutive nights during the screening period on a bladder diary (all 3 nights had to have ≥2 voids/night). Patients with other sleep disorders including known or documented diagnosis of narcolepsy, periodic limb movement disorder or moderate/severe obstructive sleep apnea (Sweetman et al., 2021), nocturia linked to urinary tract infection, neoplasm of the bladder, prostate/urethral cancer, calculi, or neurogenic voiding dysfunction were excluded. Full eligibility criteria are provided in the Supplementary Material (Data S1). The use of central nervous system active medications or moderate/strong cytochrome P450 3A4 (CYP3A4) inhibitors/inducers within 2 weeks or five half‐lives (whichever is longer) prior to randomisation until 24 h after study treatment discontinuation were prohibited. Medications to treat the primary nocturia conditions were allowed if initiated prior to trial start, and if planned to remain stable during the trial. Site investigators determined the eligibility of patients.

### 2.3 Trial procedures

Following a screening period (14–21 days), eligible patients were randomly assigned using interactive response technology (1:1) to one of two treatment sequences—daridorexant 50 mg/placebo or placebo/daridorexant. Patients received the first treatment every evening for 4 weeks (Treatment Period I), followed by a 14–21‐day washout period (considered adequate given the 8‐h half‐life of daridorexant [Muehlan et al., 2019]). Patients then crossed over to receive 4 weeks of the alternative treatment (Treatment Period II). A 5–10‐day safety follow‐up period ended the trial.

### 2.4 Endpoints

All efficacy endpoints were self‐evaluated by means of patient‐reported outcome instruments. The primary endpoint was change from baseline to Week 4 (of both treatment periods) in sTST, recorded every morning by the patient from screening through to end of Treatment Period II in a validated sleep diary questionnaire (SDQ) as described in the Supplementary Material (Data S1; Phillips‐Beyer et al., 2024).

Additional insomnia endpoints included change in sTST from baseline at Week 1, 2 and 3, weekly mean change from baseline in morning 10‐cm visual analogue scale (VAS) scores for depth and quality of sleep (Phillips‐Beyer et al., 2024), also collected daily within the SDQ, and change from baseline to Week 2 and 4 in the ISI score (Morin et al., 2011) (Data S1).

Nocturia endpoints included change from baseline to Week 1 and 4 in the number of nocturnal voids and time to first nocturnal void, assessed using the Minze Diary Pod (Minze Health, Antwerp, Belgium) (Bladt et al., 2022). The voiding diary was completed at baseline and at the beginning (first 3 days of Week 1) and end (last 3 days of Week 4) of each treatment period (Bright et al., 2014).

Endpoints to assess daytime functioning included weekly change from baseline in the IDSIQ total and domain scores (Hudgens et al., 2021; Phillips‐Beyer, Kawata, et al., 2023; Phillips‐Beyer, Olivieri, et al., 2023), and in two evening 10‐cm VAS scores for ability to function and daytime alertness (Phillips‐Beyer et al., 2024) (Data S1). Health‐related quality of life endpoints included change from baseline in the European Quality‐of‐Life five‐Dimensions three‐Level Version (EQ‐5D‐3L; Week 2 and 4) (Rabin & de Charro, 2001) and in the International Consultation on Incontinence Modular Questionnaire–Nocturia Quality of Life (ICIQ‐NQoL; Week 4) scores (Abraham et al., 2004; Mock et al., 2008) (Data S1).

At the end of each treatment period, patients reported their overall satisfaction with treatment using a numerical rating scale (0–10) and at the end of the trial, patients selected which treatment period they preferred, based on questions developed and validated with input from patients (see Data S1 Supplementary Material and Supplementary Table S1 for details).

Safety endpoints included treatment emergent adverse events (AEs), next‐morning residual effects (change from baseline in VAS‐assessed morning sleepiness) and AEs of special interest (AESI), which were falls and urinary incontinence.

### 2.5 Statistical analyses

Assuming a standard deviation (SD) of 44 min, a sample size of 50 in this crossover setting will provide at least 90% power to detect an estimated within‐patient difference of 26 min between daridorexant and placebo based on a two‐sided paired *t*‐test at the 5% significance level (Mignot et al., 2022; Zammit et al., 2020). The type I error rate was only controlled for the primary endpoint.

Efficacy and safety endpoints were analysed on all randomised patients (*N* = 60). For the primary endpoint, data were based on the mean of daily values in the 7 days preceding randomisation (baseline) and for each week during the treatment periods. Patients had to have ≥2 days of data each week to calculate a weekly mean, otherwise, the mean value was considered missing for that week (Mignot et al., 2022). Changes from baseline were analysed using a linear mixed‐effects model for repeated measures (MMRM). The model was adjusted for baseline value and included factors for treatment, treatment period and week within period, as well as treatment × week interaction. Results are reported as least‐squares (LS) mean with 95% confidence interval (CI) for change from baseline and difference to placebo. As the collection of urine during pre‐defined study nights might have an impact on the primary endpoint, a sensitivity analysis was performed excluding those days from the weekly averages.

The IDSIQ and VAS endpoints (mean of daily entries in the 7 days at each timepoint), number of nocturnal voids (mean of 3 consecutive nights at each timepoint) and ISI were analysed using the same MMRM as defined above. Time to first nocturnal void was analysed via the Kaplan–Meier approach. Treatment preference and satisfaction endpoints were analysed based on a non‐parametric sign test and paired *t*‐test, respectively. Other endpoints were summarised descriptively. All statistical analyses were done using Statistical Analysis System (SAS) software (version 9.4).

## 3 RESULTS

### 3.1 Trial population

**FIGURE 1:**
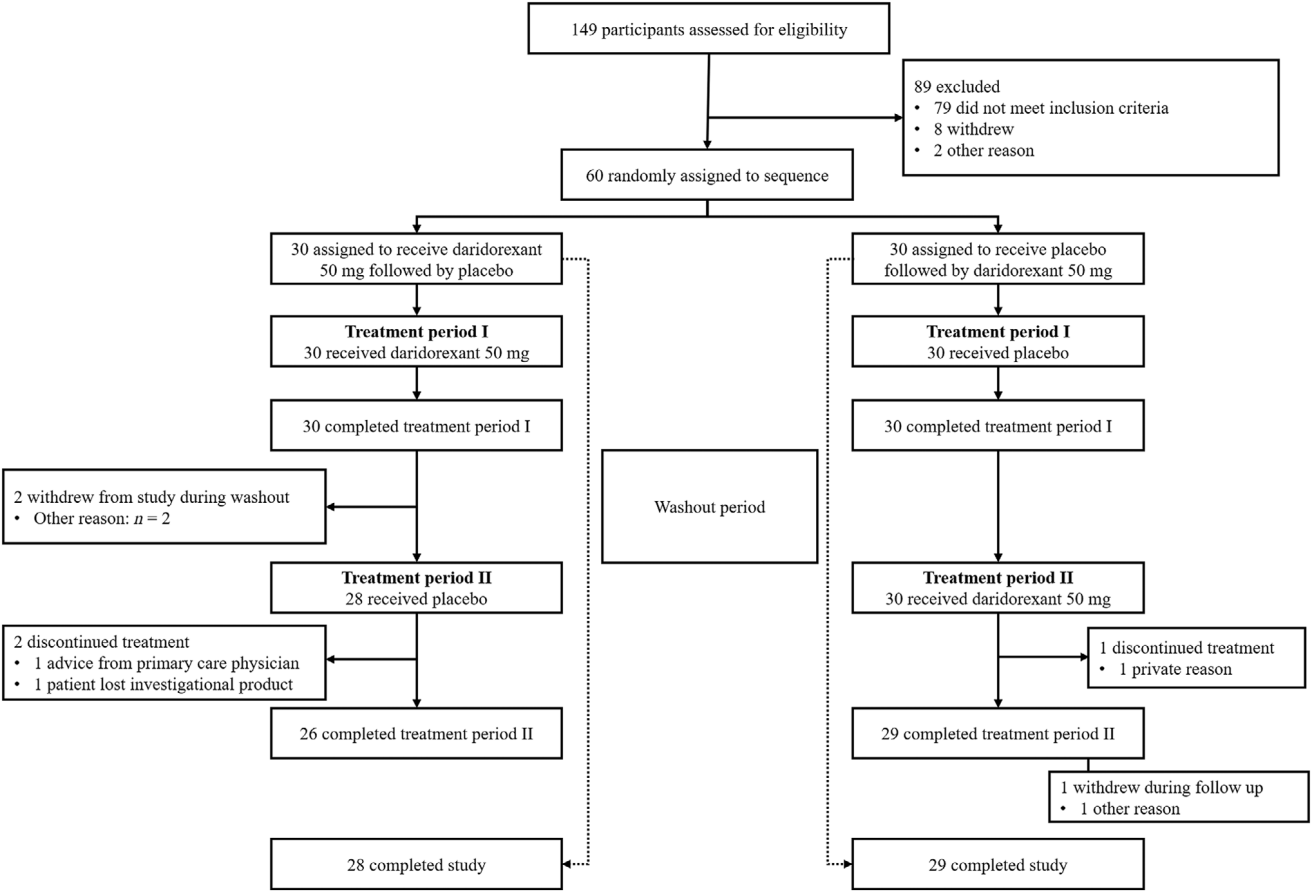
Patient disposition.

A total of 60 patients were enrolled from 18 January 2023 through 18 April 2024 and were randomly assigned to one of the two treatment sequences (*n* = 30 daridorexant 50 mg/placebo; *n* = 30 placebo/daridorexant) (Figure 1). Overall, 55 patients completed both treatment periods: all 60 completed Treatment Period I. Among patients assigned to receive daridorexant 50 mg first, two withdrew from the study during the washout period and two discontinued during Treatment Period II (placebo), and among patients assigned to receive placebo first, one discontinued treatment during Treatment Period II (daridorexant 50 mg).

Approximately equal numbers of males and females were included (Table 1). Patients had moderate/severe insomnia (mean ISI score 19.0) and clinically significant nocturia (mean of 3.6 voids/night). Significant ongoing medical conditions included hypertension (42%), type 2 diabetes (17%), urinary incontinence (17%), and benign prostatic hypertrophy (18%). Relevant concurrent medications included alpha adrenergic antagonists (12%). No hypnotics were administered.

**TABLE 1:** Patients demographics and baseline characteristics.

Characteristic	Daridorexant 50 mg/placebo, *N* = 30	Placebo/ daridorexant 50 mg, *N* = 30	Total, *N* = 60
Sex, *n* (%)			
Male	18 (60)	13 (43)	31 (52)
Female	12 (40)	17 (57)	29 (48)
Age, years, mean (SD)	64.1 (6.2)	63.8 (6.7)	64.0 (6.4)
Race, *n* (%)			
White	22 (73)	20 (67)	42 (70)
Black or African American	8 (27)	10 (33)	18 (30)
Countries, *n* (%)			
USA	20 (67)	22 (73)	42 (70)
Germany	7 (23)	6 (20)	13 (22)
Spain	3 (10)	2 (7)	5 (8)
Baseline sleep characteristic			
sTST, min, mean (SD)	367.2 (58.6)	353.2 (54.9)	360.3 (56.7)
ISI score (range 0‐28^a^), mean (SD)	19.3 (3.8)	18.8 (3.7)	19.0 (3.7)
ISI score ≤14, *n* (%)	4 (13)	5 (17)	9 (15)
ISI score 15–21, *n* (%)	16 (53)	17 (57)	33 (55)
ISI score 22–28, *n* (%)	10 (33)	8 (27)	18 (30)
VAS quality of sleep, mm^b^, mean (SD)	45.4 (21.7)	48.5 (18.8)	46.9 (20.2)
VAS depth of sleep, mm^b^, mean (SD)	47.2 (21.9)	50.2 (17.5)	48.7 (19.7)
Baseline nocturia characteristics			
Number of voids/night, mean (SD)	3.74 (1.05)	3.52 (0.89)	3.63 (0.97)
Number of voids/night, median (range)	3.8 (2.7–7.7)	3.3 (2.3–6.3)	3.5 (2.3–7.7)
Time to first void, h, median (range)	1 h 52 (1 h 20–2 h 17)	1 h 34 (1 h 21–1 h 47)	1 h 40 (1 h 26–1 h 49)
IDSIQ total score (range 0–140), mean (SD)^c^	71.1 (24.7)	62.9 (23.9)	67.0 (24.4)
VAS daytime alertness, mm^b^, mean (SD)	45.7 (21.5)	54.4 (19.5)	50.1 (20.8)
VAS ability to function, mm^b^, mean (SD)	48.6 (21.6)	56.8 (19.9)	52.7 (21.0)
ICIQ‐NQoL score (range 0–58^d^), mean (SD)	31.9 (7.7)	31.6 (7.1)	31.7 (7.4)

*Note*: some percentages do not sum to 100 because of rounding.

Abbreviations: ICIQ‐NQoL, International Consultation on Incontinence Questionnaire–Nocturia Quality of Life; IDSIQ, Insomnia Daytime Symptoms and Impact Questionnaire; ISI, Insomnia Severity Index; SD, standard deviation; VAS, visual analogue scale.

^a^ ISI Score 0–7 = absence of insomnia; 8–14 = sub‐threshold insomnia; 15–21 = moderate insomnia; score 22–28 = severe insomnia.

^b^ Higher VAS scores indicate better scores.

^c^ Lower IDSIQ scores indicate better patient‐perceived daytime functioning.

^d^ Higher ICIQ‐NQoL scores indicate a higher impact on quality of life.

### 3.2 Primary endpoint

**FIGURE 2:**
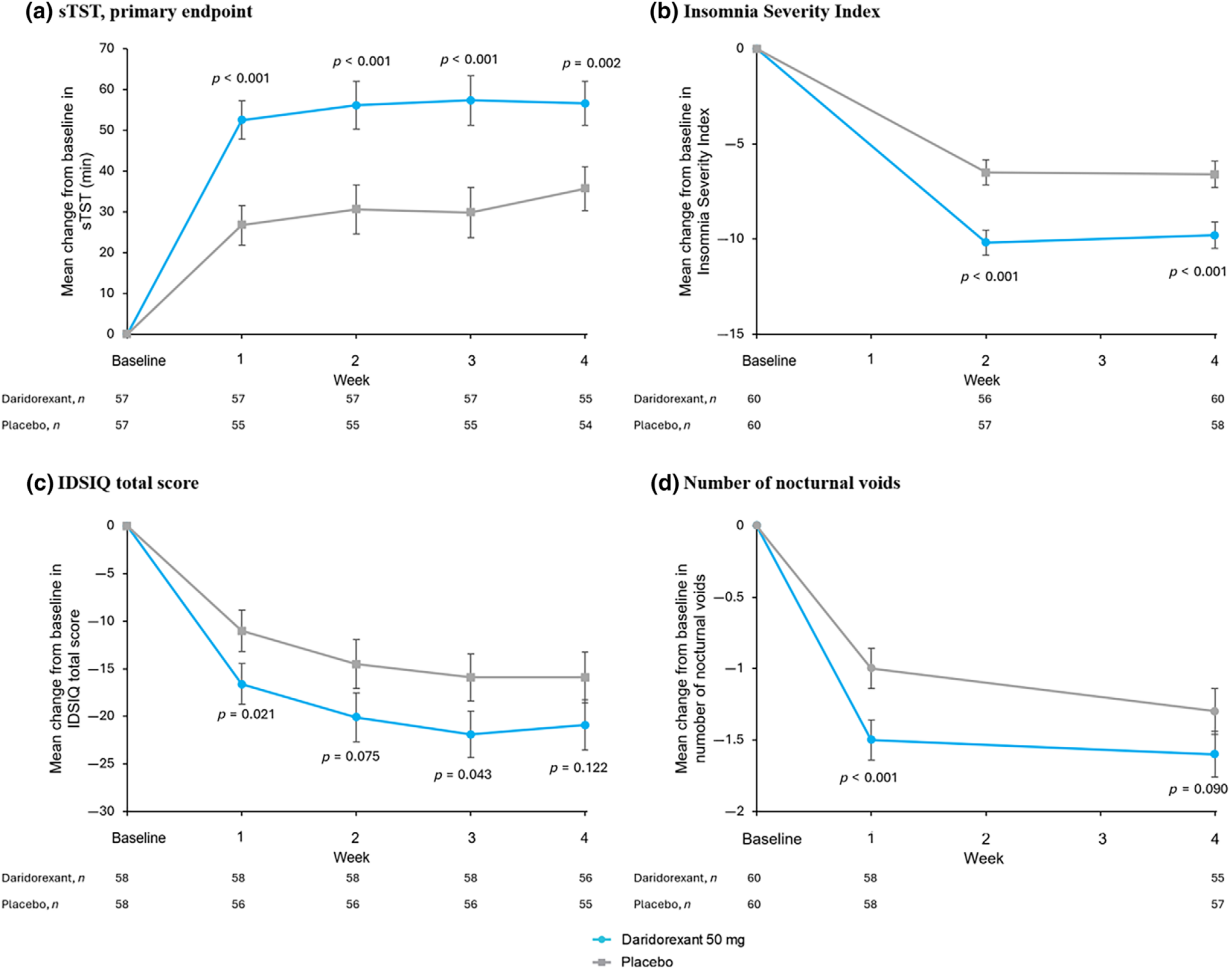
Mean changes from baseline over time in efficacy endpoints. Mean change from baseline in (a) subjective total sleep time (sTST, min), (b) Insomnia Severity Index (ISI), (c) Insomnia Daytime Symptoms and Impacts Questionnaire (IDSIQ) total score, (d) number of voids/night. sTST was collected every day in the morning and weekly averages were calculated. The ISI (scale 0–28) was collected after Week 2 and 4 of each treatment period. The IDSIQ (scale from 0 to 140) was collected every day in the evening and weekly averages were calculated. Number of voids was collected for 3 consecutive days at the beginning (Week 1) and the end of each treatment period (Week 4). Error bars show the standard error of the mean. Two‐sided *p* values shown are versus placebo.

At Week 4, the mean sTST (min) was significantly increased from baseline with daridorexant (+56.6, 95% CI 46.0–67.2 min) compared to placebo (+35.7, 95% CI 25.0–46.4 min), with a LS mean difference of +20.9 (95% CI 8.0–33.7 min; *p* = 0.002; Figure 2). Significant increases in mean sTST with daridorexant versus placebo were also seen at Weeks 1, 2 and 3. Sensitivity analyses showed similar results (Table S2).

### 3.3 Additional insomnia endpoints

The ISI score decreased (improved) from baseline on both treatments with larger decreases on daridorexant versus placebo at Week 2 (−10.2 versus −6.5) and Week 4 (−9.8 versus −6.6) (Figure 2). The LS mean difference between treatments was statistically significant at both weeks (Week 2: −3.7, 95% CI −5.1 to −2.3, *p* < 0.001; Week 4: −3.3, 95% CI −4.7 to −1.8, *p* < 0.001).

The proportion of patients with a decrease of ≥6 points in the ISI score from baseline was numerically greater on daridorexant than placebo at both timepoints (Week 2: 79% versus 53%; Week 4: 80% versus 53%) as was the proportion of patients with an ISI score of <7 indicating no remaining clinically significant insomnia (Week 2: 46% versus 18%; Week 4: 43% versus 21%).

**FIGURE 3:**
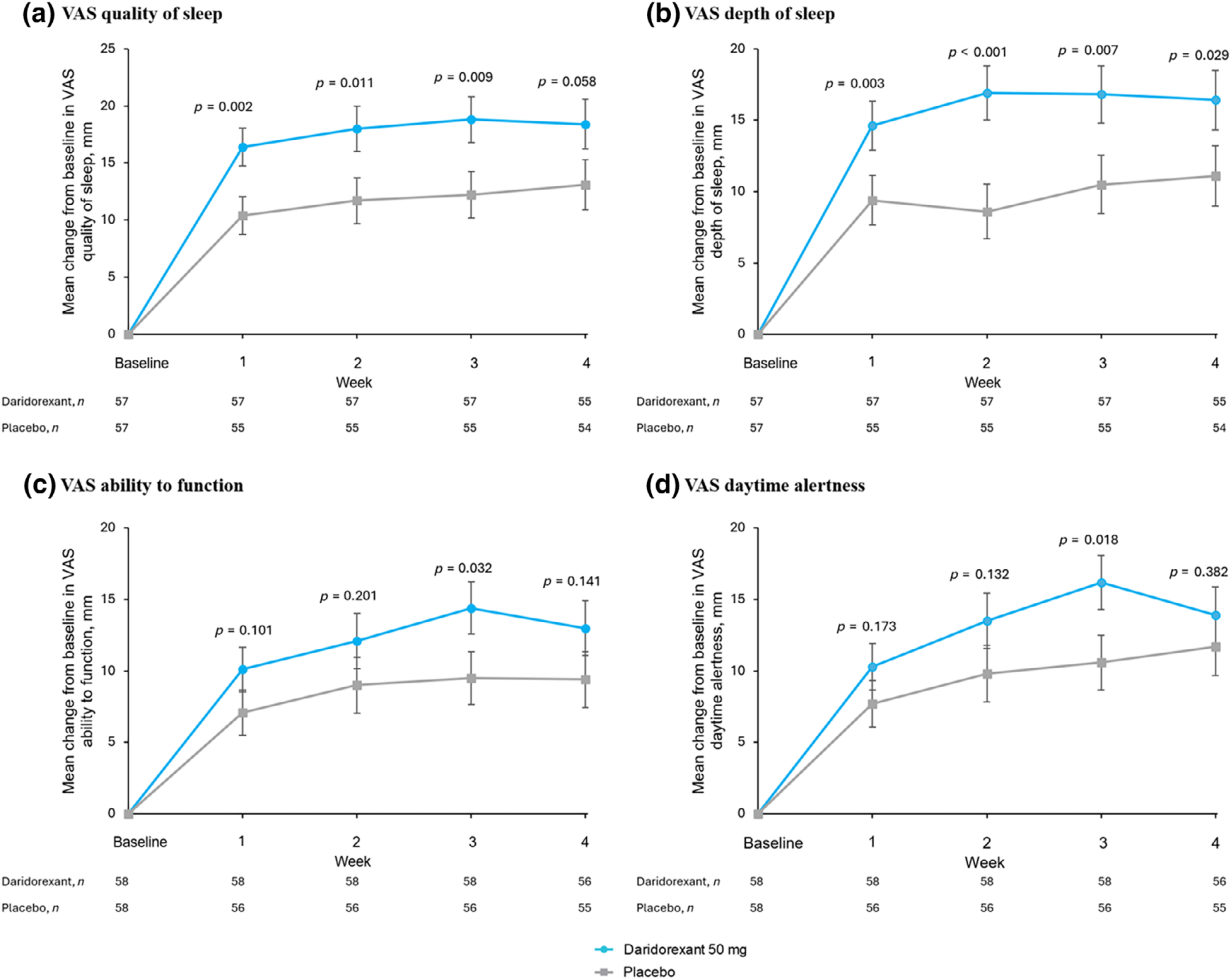
Mean change from baseline over time on subjective assessment of sleep and daytime symptoms assessed by visual analogue scale (VAS). Weekly mean change from baseline in the 100‐mm VAS (a) quality of sleep, (b) depth of sleep, (c) ability to function and (d) daytime alertness. Higher VAS scores indicate better scores. Error bars show the standard error of the mean. Two‐sided *p* values shown are versus placebo.

Increases (i.e., improvements) in VAS scores for quality and depth of sleep were consistently larger with daridorexant than placebo (Figure 3; Table S3), with statistical significance (*p* < 0.05) at all four weekly timepoints for both endpoints (with one exception, quality of sleep at Week 4; *p* = 0.058).

### 3.4 Nocturia endpoints

Daridorexant decreased the number of voids per night from baseline to Week 1 (LS mean −1.5, 95% CI −1.8 to −1.2) and Week 4 (LS mean −1.6, 95% CI −2.0 to −1.3) by a greater magnitude than placebo (Week 1: LS mean −1.0, 95% CI[−1.2 to −0.7]; Week 4: LS mean −1.3, 95% CI −1.6 to −1.0). The LS mean difference in voids per night between daridorexant and placebo was −0.6 (95% CI −0.9 to −0.3; *p* < 0.001) at Week 1 and −0.3 (95% CI −0.7 to +0.1; *p* = 0.090) at Week 4 (Figure 2).

Voiding at least twice a night is considered clinically meaningful (Tikkinen et al., 2010; Yu et al., 2006). At Week 1, 47% of patients on daridorexant reported <2 voids/night compared to 19% on placebo, increasing to 53% versus 32%, respectively at Week 4 (Table S4).

The median time to first void increased from 1.67 h (95% CI 1.44–1.82) at baseline to 2.36 h (1.95–2.95) on daridorexant and 1.85 h (1.43–2.14) on placebo at Week 1 and to 2.46 h (1.93–2.77) on daridorexant and to 2.09 h (1.70–2.71) on placebo at Week 4. The median difference between daridorexant and placebo was +31 min (*p* = 0.0027) and +23 min (*p* = 0.2026) at Week 1 and 4, respectively (Figure S1).

### 3.5 Daytime functioning and quality of life

The IDSIQ total scores decreased (improved) over time, with numerically greater improvements observed with daridorexant versus placebo (Figure 2; Table S5). LS mean changes from baseline were significantly greater with daridorexant at Week 1 (*p* = 0.021) and Week 3 (*p* = 0.043). The mean reduction from baseline in total IDSIQ score on daridorexant at Week 1 of 16.8 virtually equated to the meaningful score difference of 17 that has been determined based on what patients regard as a clinically meaningful within‐patient change (Phillips‐Beyer, Kawata, et al., 2023), and exceeded this threshold at all subsequent timepoints; on placebo, mean reduction in total IDSIQ scores remained below 16 units at all timepoints. A similar pattern of effect over time was observed for the IDSIQ domain scores (Table S5).

The VAS scores for ability to function and daytime alertness also increased (improved) by a greater magnitude on daridorexant than placebo at all timepoints, without reaching statistical significance (except Week 3, *p* < 0.05 for both endpoints; Figure 3; Table S3).

Reductions (improvements) in the ICIQ‐NQoL score from baseline to Week 4 were numerically greater on daridorexant than placebo (Table S6). The EQ‐5D‐3L scores at baseline were high (mean [SD] 0.90 [0.10]). At Week 2, scores slightly increased (improved) on daridorexant and slightly decreased (worsened) on placebo; at Week 4, scores increased slightly from baseline for both treatments (Table S7).

### 3.6 Patient satisfaction and treatment preference

A significantly higher mean satisfaction score was reported by patients at the end of treatment with daridorexant (7.0, 95% CI 6.3–7.6) versus placebo (5.3, 95% CI 4.3–6.2), with an LS mean difference of 1.7 (95% CI 0.7–2.7; *p* = 0.001). At the end of the trial, more patients expressed a preference for daridorexant than placebo (74% versus 26%; odds ratio 2.9, 95% CI 1.59–5.16; *p* = 0.0004).

### 3.7 Safety

Treatment emergent AEs (TEAEs) were reported in 15 patients (25%) on daridorexant and 10 patients (17%) on placebo (Table 2). No serious AEs, AEs leading to study drug discontinuation or deaths were reported during the trial. No AESIs were reported on daridorexant; on placebo, two patients reported an AESI (fall in one; urinary incontinence episode in one). Other relevant TEAEs included somnolence (reported by one patient while receiving daridorexant and a second patient while receiving placebo), fatigue in three patients while receiving daridorexant, and hangover effect in two patients on daridorexant.

**TABLE 2:** Adverse events in the safety analysis population.

Preferred term	Daridorexant, *N* = 60	Placebo, *N* = 60	Total, *N* = 60
Patients with ≥1 adverse event, *n* (%)	15 (25)	10 (17)	22 (37)
Adverse events reported in ≥2 patients in any treatment, *n* (%)			
Dry mouth	3 (5)	2 (3)	4 (7)
Fatigue	3 (5)	0	3 (5)
Nausea	2 (3)	0	2 (3)
Hangover	2 (3)	0	2 (3)
Adverse events of special interest	0	2 (3)	2 (3)
Fall	0	1 (2)	1 (2)
Incontinence	0	1 (2)	1 (2)

*Note*: adverse events during the washout period are assigned to the treatment group that was administered prior to the washout period.

Mean VAS scores for morning sleepiness improved over time on both treatments, more so on daridorexant versus placebo at each week (Table S8).

## 4 DISCUSSION

This placebo‐controlled trial shows that, in patients with chronic insomnia and nocturia, daridorexant improves subjective sleep and nocturia symptoms simultaneously, and decreases daytime impairment. These effects were observed from Week 1 and maintained for the 4‐week treatment duration. Patients identified their preference in favour of daridorexant while being unaware of the treatment assignment and indicated a high level of treatment satisfaction on daridorexant. No new safety concerns were identified, and no falls or injuries were observed on daridorexant.

Patients were selected based on subjective self‐assessment of their insomnia symptoms reflecting the real‐life situation to which general practitioners will be confronted. Nevertheless, despite not using stringent polysomnography (PSG)‐based sleep criteria, the baseline insomnia characteristics of the population (mean sTST, ISI, IDSIQ) were overall similar to those of previous insomnia studies (Mignot et al., 2022), corresponding to moderate/severe chronic insomnia disorder. Mean improvements from baseline in sTST in patients treated with daridorexant exceeded the clinically relevant minimum difference of 55 min (Phillips‐Beyer et al., 2024) with a virtually identical placebo‐corrected treatment effect as reported in previous insomnia studies (Mignot et al., 2022). Although the baseline ISI in our trial was similar to that recorded in insomnia trials (Mignot et al., 2022), compared to these trials, we report a larger improvement in the ISI on daridorexant, with twice as many patients improving by ≥6 units, the threshold of clinical relevance (Yang et al., 2009). This higher perceived reduction in insomnia severity may, in part, be attributed to the early improvement in time to first nocturnal void after sleep onset, an important indicator of overall sleep quality (Bliwise et al., 2015). A shorter time to first void is associated with increased daytime dysfunction and decreased sleep quality, quantity and/or sleep efficiency (Bliwise et al., 2015) and indeed, we observed treatment effects as early as Week 1 in sTST, VAS quality and depth of sleep, and the IDSIQ total/domain scores. Additionally, the potential of daridorexant to reduce the number of nocturnal voids, long wake bouts and hyperarousal may further contribute to this effect (Di Marco et al., 2023; Di Marco et al., 2024).

The improvements in nocturia symptoms observed with daridorexant are consistent with the benefits observed with usual treatments of nocturia (Han et al., 2018; Kaminetsky et al., 2018). One may speculate that the improvements we observed are a consequence of the improvement in sleep. However, based on a potential role of the orexin system in micturition (Kobayashi et al., 2009; Serefko et al., 2025), daridorexant may also have an effect on nocturia symptoms independent of sleep. It has been shown that orexin‐A in the spinal cord activates the micturition reflex in normal rats (Kobayashi et al., 2009) and that antagonism of orexin 2 receptors in rats with induced overactive bladder restores the abnormal levels of overactive bladder markers (Serefko et al., 2025). Furthermore, clinical data suggest that orexin antagonists may increase bladder capacity by prolonging the interval between bladder contractions, resulting in a decrease in the number of nocturnal voids (Togo et al., 2024).

Daytime functioning is negatively affected in patients who experience nocturia and/or insomnia (Bliwise et al., 2019; Kyle et al., 2010) and the baseline IDSIQ reported here indicates a level of daytime impairment similar to that reported in patients with insomnia (Mignot et al., 2022). Improvements in the IDSIQ scores were seen as early as Week 1 of treatment and were maintained throughout the treatment period. The mean reduction (improvement) of the total score exceeded the validated threshold for meaningful improvement in an individual (Phillips‐Beyer, Kawata, et al., 2023), indicating the clinical relevance of the findings.

The crossover design of the trial allowed the assessment of patient's perspective on their treatment preference. This is a relevant question given the difficulties in treatment choice, poor adherence (Jayadevappa et al., 2015; Veenboer & Bosch, 2014) and the need for long‐term treatment in both conditions. Although substantial placebo effects were observed on insomnia, nocturia, and daytime functioning endpoints, patients expressed a large preference for daridorexant compared to placebo. This, plus the high level of patient satisfaction with daridorexant, may help patients maintain good adherence to long‐term therapy. Longer studies in real‐life situations would need to confirm adequate adherence.

The safety profile of daridorexant is well established in the treatment of insomnia and no new safety signals were reported here. Importantly, in a study population that is prone to an increased risk of falls and fractures (Nakagawa et al., 2010), we report no increased risk in falls with daridorexant, consistent with previous studies in insomnia (Mignot et al., 2022). In addition, the reduction of nocturnal voids and increased time to first void were not associated with any increase in urinary incontinence.

Strengths of the trial include the placebo‐controlled, crossover design, with patients as their own matched control, the inclusion of both male and female patients and 30% Black/African American patients (which represents more than double the proportion of the Black/African American population in the United States (United States Census Bureau, 2021)) and the patient selection based on criteria that identified patients with moderate/severe chronic insomnia and clinically relevant nocturia irrespective of cause. The trial focuses on patient‐reported assessments to reflect the importance of the patients’ experience on the impact of the diseases on daily life and how daridorexant is able to affect it. Nevertheless, the trial is not without limitations, which include the short‐term treatment duration and the lack of objective sleep measures such as PSG. Although subjective assessments add significant value from both a clinical and practical perspective, and the validity of sTST (the primary endpoint) in providing clinically meaningful information has been documented (Phillips‐Beyer et al., 2024), discrepancies between subjective and objective sleep assessments (sleep state misperception) are common (Castelnovo et al., 2019). PSG could have offered insights into temporal variations in sleep patterns including sleep architecture, and the time to return to sleep and could also have been useful to exclude other sleep disorders. However, considering that this population frequently wakes up during the night to use the bathroom, performing PSG was considered impractical.

## 5 CONCLUSION

Our results provide evidence of the benefit of daridorexant, a DORA, in patients with chronic insomnia and nocturia, with efficacy on symptoms of both conditions, improvement in daytime functioning, and a good safety and tolerability profile.

## AUTHOR CONTRIBUTIONS

**Katharina Lederer:** Investigation; writing – review and editing; visualization. **Heike Benes:** Investigation; writing – review and editing; visualization. **Alan Fine:** Investigation; writing – review and editing; visualization. **Sylvia Shoffner:** Investigation. **Sandro Bacchelli:** Investigation; writing – review and editing; visualization. **David Castro Diaz:** Investigation; writing – review and editing; visualization. **Jose Emilio Batista:** Investigation; writing – review and editing; visualization. **Racheal Rowles:** Conceptualization; methodology; writing – original draft; visualization. **Tobias Di Marco:** Conceptualization; methodology; writing – review and editing; writing – original draft; visualization. **Michael Meinel:** Conceptualization; methodology; writing – review and editing; writing – original draft; formal analysis; validation; data curation; visualization.

## FUNDING INFORMATION

The studies were funded by Idorsia Pharmaceuticals Ltd.

## CONFLICT OF INTEREST STATEMENT

Heike Benes has received speaker fees from Idorsia Pharmaceuticals and participated in advisory boards from Idorsia Pharmaceuticals and Takeda. Jose Emilio Batista has received CME grants from Lacer Italfarmaco, Astellas Europe, Hollister and Coloplast. Michael Meinel, Racheal Rowles and Tobias Di Marco were all employees of Idorsia Pharmaceuticals Ltd at the time of the study. Alan Fine, David Castro Diaz, Katharina Lederer, Sandro Bacchelli, and Sylvia Shoffner have no conflicts of interest to disclose.

## ETHICS STATEMENT

The study was conducted in accordance with the International Conference on Harmonisation guidelines for Good Clinical Practice and principles of the Declaration of Helsinki.

## PATIENT CONSENT STATEMENT

All subjects gave written informed consent.

## CLINICAL TRIALS REGISTRATION

ClinicalTrials.gov identifier: NCT05597020.

## Supporting information


**Data S1.**Supplementary Material.

## Data Availability

In addition to Idorsia's existing clinical trial disclosure activities, the company is committed to implementing the Principles for Responsible Clinical Trial Data Sharing jointly issued by the European Federation of Pharmaceutical Industries and Associations (EFPIA) and the Pharmaceutical Research and Manufacturers of America (PhRMA). Requests for data sharing, of any level, can be directed to clinical‐trials‐disclosure@idorsia.com for medical and scientific evaluation.
