# Supplementary material for: A randomised crossover trial of daridorexant for the treatment of chronic insomnia and nocturia
Source: J Sleep Res. 2025 Mar 13;34(6):e70002. doi: 10.1111/jsr.70002 (PMC12592811; doi:10.1111/jsr.70002)
Supplement: Supplementary file 1 — Data S1.Supplementary Material. [file JSR-34-e70002-s001.docx]

**Supplementary Material**

**A randomized cross-over trial of daridorexant for the treatment of chronic insomnia and nocturia**

Katharina Lederer, Heike Benes, Alan Fine, Sylvia Shoffner, Sandro Bacchelli, David Castro Diaz, Jose Emilio Batista, Racheal Rowles, Tobias Di Marco, Michael Meinel

**Full inclusion and exclusion criteria**

**Inclusion criteria**

Subjects must meet all the following inclusion criteria:

1. Signed and dated ICF prior to any study-mandated procedure.
2. Male or female subjects ≥ 55 years old at the time of signing the informed consent form (ICF).
3. Insomnia complaints for ≥ 3 months prior to Visit 1.
4. ISI^©^ score ≥ 13 at Visit 1.
5. Diagnosis of nocturia as per International Continence Society:
   - Waking to pass urine during the main sleep period.
   - The first nocturia episode must be preceded by sleep. Subsequent nocturia episodes must be followed by the intention of getting back to sleep.
6. Nocturia severity: on average ≥ 3 nocturnal voidings per night reported by the subjects for ≥ 1 month prior to Visit 1.
7. Average ≥ 2.6 nocturia episodes reported on the bladder diary per night over a period of 3 consecutive nights following Visit 1. None of the 3 nights can have less than 2 voidings.
8. Ability to communicate well with the investigator, to understand the study requirements, and judged by the investigator to be alert and oriented to person, place, time, and situation.

**Exclusion criteria**

Subjects must not meet any of the following exclusion criteria:

1. Woman of childbearing potential, pregnant or planning to become pregnant.
2. Planned travel across ≥ 3 time zones during study.
3. Any of the following conditions related to suicidality:
   - Any suicidal ideation with intent, with or without a plan, at Screening, i.e., answering “Yes” to questions 4 or 5 on the suicidal ideation section of the lifetime (screening visit) version of the C-SSRS^©^.
   - History of suicide attempt on the suicidal behavioral section of the lifetime version of the C-SSRS^©^ (screening visit).
4. Regular caffeine consumption after 4pm.
5. Unable to refrain from smoking during the night.
6. Known and documented diagnosis of narcolepsy, periodic limb movement disorder, moderate to severe obstructive sleep apnea, restless legs syndrome, circadian rhythm sleep-wake disorder, or rapid eye movement (REM) sleep behavior disorder.
7. Known and documented diagnosis of Type 1 diabetes mellitus, uncontrolled Type 2 diabetes mellitus, central or nephrogenic diabetes insipidus, and primary/secondary polydipsia within the last 6 months prior to Visit 1.
8. Known and documented nocturia linked to urinary tract infection, neoplasms of bladder, prostate or urethral cancer, bladder or urethral calculi, or neurogenic voiding dysfunction within the last 6 months prior to Visit 1.
9. Any signs or symptoms of active, ongoing infection judged to be clinically relevant by the investigator.
10. Known and documented diagnosis of severe compromised respiratory function (e.g., chronic obstructive pulmonary disease).
11. Known and documented moderate to severe hepatic impairment.
12. Known hypersensitivity to the investigational treatment, any of its excipients or drugs of the same class.
13. Treatment with forbidden medications including moderate or strong CYP3A4 inhibitors and inducers 2 weeks prior to Visit 2.
14. Treatment with another investigational treatment or participation in another clinical trial up to 3 months prior to Visit 2.
15. Any known factor or disease that might interfere with treatment compliance, study conduct, or interpretation of the results, such as drug or alcohol dependence or psychiatric disease.
16. Known concomitant life-threatening disease with a life expectancy < 12 months.
17. Ongoing CBT-I at Visit 1 or planned start during the study.

**Sleep diary (eDiary)**

The sleep diary (also called eDiary) was uploaded onto an electronic hand-held device and given to the patients at screening (Visit 1). Sleep diaries were available in the patient’s language and were required to be completed daily from Visit 1 until Visit 8 (end of Treatment Period II). The self-administered sleep diary included a morning and evening questionnaire which collected information on self-reported sleep characteristics such as sTST and five visual analog scales (VAS) [1].

**A) Morning and evening questionnaires** [1].

Data for the primary endpoint, sTST, were collected as part of the morning questionnaire, where patients were instructed to give their best estimate to answer the question (Q9): “*In total, how long did you sleep last night?”* In addition, the morning and evening questionnaires collected information on other self-reported quantitative sleep characteristics (sleep induction and maintenance), napping, bedtime, and timing of study treatment intake.

**Morning questionnaire**

Q1. *Did you take your study medication last night*? Yes or No

Q2. *If yes, at what time did you take your study medication last night?*

Q3. *What time did you get into bed?* (Write the time that you got into bed. This may not be the time that you began “trying” to fall asleep.)

Q4. *What time did you try to go to sleep?* (Record the time that you began “trying” to fall asleep)

Q5. *How long did it take you to fall asleep?* (Beginning at the time you wrote in question 4, how long did it take you to fall asleep?)

Q6. *How many times did you wake up, not counting your final awakening?* (How many times did you wake up between the time you first fell asleep and your final awakening?)

Q7. *In total, how long did these awakenings last?* (What was the total time you were awake between the time you first fell asleep and your final awakening? For example, if you woke 3 times for 20 minutes, 35 minutes, and 15 minutes, add them all up: 20+35+15=70 min or 1h 10 min).

Q8. *What time was your final awakening?* (Record the last time you woke up in the morning)

Q9. *In total, how long did you sleep last night?* (This should just be your best estimate, based on when you went to bed and woke up, how long it took you to fall asleep, and how long you were awake. You do not need to calculate this by adding and subtracting; just give your best estimate.)

Q10. *What time did you get out of bed for the day?* (What time did you get out of bed with no further attempt at sleeping? This may be different from your final awakening time (e.g., you may have woken up at 6:35 a.m. but did not get out of bed to start your day until 7:20 a.m.)

**Evening questionnaire**

Q1. *How many times did you nap or doze?* (A nap is a time you decided to sleep during the day, whether in bed or not in bed. “Dozing” is a time you may have nodded off for a few minutes, without meaning to, such as while watching TV. Count all the times you napped or dozed at any time from when you first got out of bed in the morning until you got into bed again at night.)

Q2. *In total, how long did you nap or doze?* (Estimate the total amount of time you spent napping or dozing, in hours and minutes. For instance, if you napped twice, once for 30 minutes and once for 60 minutes, and dozed for 10 minutes, you would answer “1 hour 40 minutes.”)

**B) Visual analog scales** [1]

The VAS collected information on quality of sleep, depth of sleep, morning sleepiness, daytime alertness, and daytime ability to function by asking the patients to report their feelings by placing a mark on a VAS. Self-reported quality of sleep, depth of sleep, and morning sleepiness were assessed in the morning. Self-reported daytime alertness and daytime ability to function were assessed in the evening.


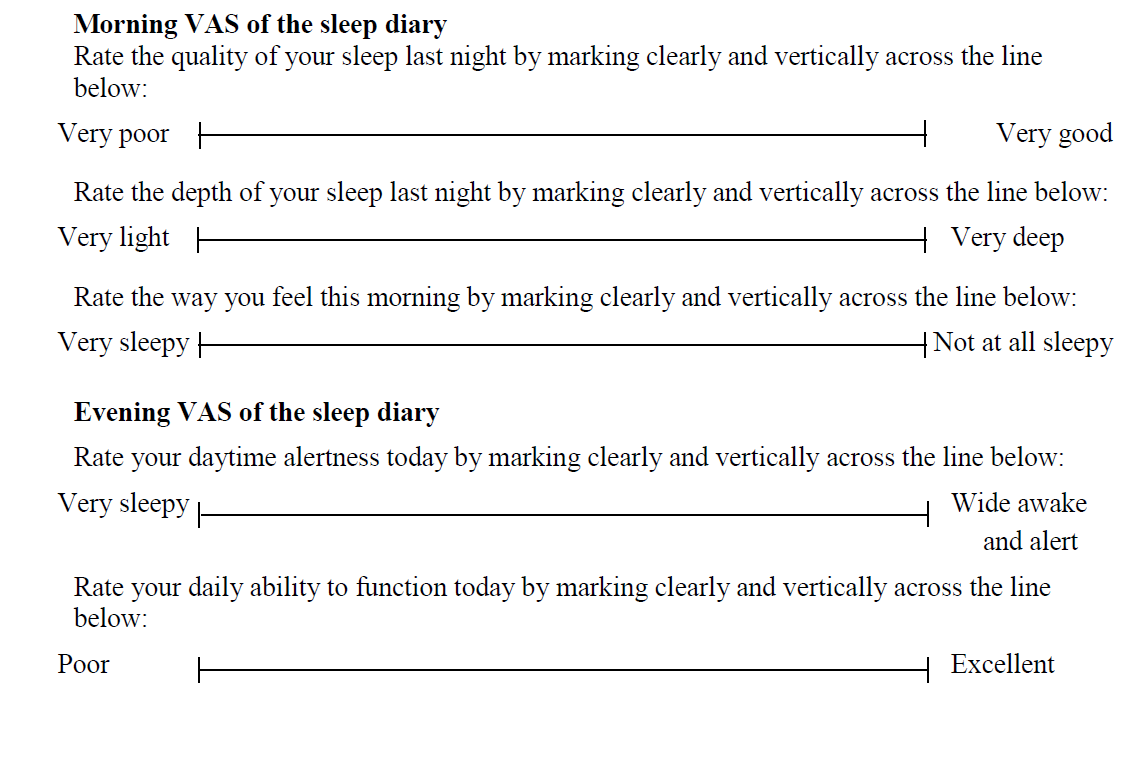
 **Insomnia Severity Index^©^** [2]

The ISI^©^ assessed the severity of a patient’s insomnia by scoring the severity of sleep onset and sleep maintenance difficulties and any insomnia-related interference with daytime functioning. The assessment is on a 5-point scale (0–4), where the composite score was obtained by summing the seven rated dimensions measuring the patient’s perception of his or her insomnia. A score of 15–21 indicates a moderate level of insomnia and a score of 22–28 indicates severe insomnia. An ISI^©^ total score <10 indicates that the patient’s subjectively rated insomnia symptoms, daytime impairment, and quality of life have improved to the minimal-to-none range [2]. The ISI^©^ was completed by the patient on the hand-held device at screening visit (Visit 1), and Weeks 2 and 4 of each treatment period. The ISI^©^ questionnaire used in this trial used a recall period of 2 weeks.

**Insomnia Daytime Symptoms and Impacts Questionnaire** [3, 4]

The IDSIQ was programmed on the electronic hand-held device in the patient’s language and was required to be completed every day in the evening before the evening sleep diary by the patient from screening (Visit 1) until end of Treatment Period II (Visit 8) with a recall period of ‘today’. The IDSIQ is a validated questionnaire that has been developed to assess daytime functioning in patients with insomnia disorder [3, 4] and in patients with insomnia disorder and comorbid nocturia [5]. It contains 14 different questions grouped into three domains each representing the main daytime symptoms and impacts of insomnia on sleepiness (four items; scores 0-40), alert/cognition (six items; scores 0-60) and mood (four items; scores 0-40). Each question is scored by the patient on an 11-point numerical scale (from 0 to 10) with lower scores denoting better daytime functioning. The total score ranges from 0 to 140.

**Minze diary** [6]

The Minze Diary Pod consists of a voiding pod and a voiding diary and uses a derivation from the ICIQ bladder diary [6]. The Minze Diary Pod was required to be completed during the day and preferably started in the morning meaning that the completion started only in the morning after Visit 1 to assess eligibility. Furthermore, the Minze Diary Pod was completed in the morning, at the beginning (first 3 days of Week 1) and end (last 3 days of Week 4) of each treatment period.

**EQ-5D-3L** [7]

The EQ-5D-3L, a widely used generic instrument to assess health-related quality of life irrespective of disease [7], was completed at baseline and at Weeks 2 and 4 in both treatment periods. It measures five dimensions of health i.e. mobility, self-care, usual activities, pain/discomfort, and anxiety/depression [8], with each dimension having three response levels of severity (no problems, some problems, extreme problems). It has no recall period, with the measure being a snap-shot assessment on the day it is completed. The overall score ranges from 0 (a state as bad as being dead) to 1 (full health). Depending on the box checked by the subject a number is assigned to each dimension resulting in a 5-digit number combination (e.g., a completely healthy subject would have a score of 11111).

**ICIQ-NQoL** [9]

The ICIQ-NQoL, an adaptation of the NQoL [9], to assess specifically the impact of nocturia on quality of life in men and women, was completed by patients at baseline and at the end of each treatment period. It consists of 13 items covering daytime and nighttime impact of nocturia. The recall period is 4 weeks. The overall ICIQ-NQoL score is calculated by summing the first 12 items (ranging from 0 to 4), resulting in an overall score that ranges from 0–48 where lower scores indicate an increase in in quality of life.

**Development and validation of the patient satisfaction and treatment preference survey**

A cross-sectional qualitative observational study assessed the content validity of a four-item web-based survey designed to collect patient feedback on treatment satisfaction and preferences among patients with insomnia disorder and nocturia. In total, 20 adults (mean age 60.9 years, 65% female, 85% White) with self-reported symptoms reflecting the DSM-5 criteria for insomnia disorder and nocturia (at least two nocturnal voids per night within the last 30 days prior to screening) from the US were recruited between May – Aug 2022. All patients were willing and able to read, speak and understand English. Data collection involved 20 one-to-one web-based interviews, completed in 60-90 minutes.

Subjects were asked to describe their impressions of the questionnaire which comprised a narrative text and four questions (Table S1). The majority of subjects found the questionnaire easy to understand (n=17/18, 94%) and clear (n=15/18, 83%). Most understood the narrative text (n=17/20; 85%) and the items (range n=16/19, 84% [Question 1] to n=20/20, 100% [Questions 3 and 4]). Several subjects identified words or phrases that may be unclear across components of the questionnaire. These included “overall feelings” (Questions 1 and 2; n=4 and 2, respectively), description of the 11-point numeric rating scale (Question 3, n=3), “free text box” (Narrative text, n=1), and “while on treatment” (Question 2, n=1). For each component of the questionnaire, between 20% [Question 4] and 60% [Question 3] of subjects provided suggestions for improvement, as summarized in Table S1.

Table S1 Summary of suggested revisions to the patient satisfaction and treatment preference survey

| **Component** | **Original Text** | **Suggested Changes Clean** |
| --- | --- | --- |
| Narrative Text | “A fundamental part of this clinical trial is to understand the subject’s perception while being on treatment. This means that you will be asked to provide a written description of your experience in this study with as much details as possible. Please note that this is a free text box meaning there are no specific rules on the topics (e.g., your social life, your wellbeing or anything else that is important to you) thus they can be positive, negative or neutral.” | “As part of this clinical trial, Idorsia would like to understand your experience **while on study treatment**. Your thoughts are important to Idorsia. Please give a detailed written description in response to the questions below. Text boxes are provided for you to write on any topic (for example, your social life, your well-being, or anything else that is important to you).” |
| Question 1 | “Please provide a description (i.e., in a few sentences) of your overall feeling before entering the study.” | **Before** starting the clinical trial, how did insomnia and nocturia impact your daily life? Please describe in a few sentences.” |
| Question 2 | “Please provide a description (i.e., in a few sentences) of your overall feeling while on treatment”. | “How did the impact on your daily life from insomnia and nocturia change **during** the clinical trial? Please describe in a few sentences.” |
| Question 3 | “Rate your satisfaction with the treatment by marking clearly and vertically across the line below.” | “Please mark the number that best describes your overall satisfaction with the study treatment **during** the clinical trial. (Mark one number only)”  Response options: number scale 0 (not at all satisfied) to 10 (extremely satisfied). |
| Question 4 | “Please specify which treatment period did you prefer”. | “Which treatment period did you prefer?”   - Treatment Period I - Treatment Period II. |

**Table S2 Change from baseline in sTST at Weeks 1, 2, 3 and 4**

| **sTST, min** | **Daridorexant 50 mg n = 60** | **Placebo n = 60** |
| --- | --- | --- |
| Change from baseline to Week 1  n | 57 | 55 |
| LSM change (95% CI) | 52.5 (43.1, 61.9) | 26.7 (17.1, 36.3) |
| LSM difference to placebo (95% CI) | 25.8 (15.0, 36.6) | NA |
| p-value (two-sided) | <0.001 | NA |
| Change from baseline to Week 2  n | 57 | 55 |
| LSM change (95% CI) | 56.1 (44.5, 67.7) | 30.6 (18.8, 42.4) |
| LSM difference to placebo (95% CI) | 25.5 (11.0, 40.0) | NA |
| p-value (two-sided) | <0.001 | NA |
| Change from baseline to Week 3  n | 57 | 55 |
| LSM change (95% CI) | 57.3 (45.2, 69.3) | 29.8 (17.5, 42.1) |
| LSM difference to placebo (95% CI) | 27.4 (12.1, 42.8) | NA |
| p-value (two-sided) | <0.001 | NA |
| Change from baseline to Week 4  n | 55 | 54 |
| LSM change (95% CI) | 56.6 (46.0, 67.2) | 35.7 (25.0, 46.4) |
| LSM difference to placebo (95% CI) | 20.9 (8.0, 33.7) | NA |
| p-value (two-sided) | 0.002 | NA |
| **Sensitivity analysis^a^** |  |  |
| Change from baseline to Week 1  n | 57 | 55 |
| LSM change (95% CI) | 49.3 (38.8, 59.9) | 23.5 (12.8, 34.3) |
| LSM difference to placebo (95% CI) | 25.8 (12.7, 38.9) | NA |
| p-value (two-sided) | <0.001 | NA |
| Change from baseline to Week 2  n | 57 | 55 |
| LSM change (95% CI) | 54.9 (43.5, 66.3) | 29.7 (18.1, 41.2) |
| LSM difference to placebo (95% CI) | 25.2 (10.7, 39.7) | NA |
| p-value (two-sided) | <0.001 | NA |
| Change from baseline to Week 3  n | 57 | 55 |
| LSM change (95% CI) | 58.1 (46.4, 69.7) | 29.0 (17.1, 40.8) |
| LSM difference to placebo (95% CI) | 29.1 (14.2, 44.0) | NA |
| p-value (two-sided) | <0.001 | NA |
| Change from baseline to Week 4  n | 55 | 54 |
| LSM change (95% CI) | 60.0 (48.5., 71.6) | 34.7 (23.0, 46.5) |
| LSM difference to placebo (95% CI) | 25.3 (10.4, 40.2) | NA |
| p-value (two-sided) | 0.001 | NA |

LSM change from baseline and LSM difference to placebo are presented as LSM (95% CI). CI, confidence interval; LSM, least square mean; NA, not applicable; sTST, subjective total sleep time. Linear mixed-effects model for repeated measures: change from baseline in sTST = treatment group + baseline sTST + week + period + treatment group x week. ^a^Sensitivity analysis does not consider the days of urine collection.

**Table S3 Change from baseline in depth of sleep, quality of sleep, daytime alertness and ability to function, as measured by VAS, at Weeks 1, 2, 3 and 4**

|  | **Daridorexant 50 mg n = 60** | **Placebo n = 60** |
| --- | --- | --- |
| **VAS depth of sleep, mm** |  |  |
| Change from baseline to Week 1 |  |  |
| n | 57 | 55 |
| LSM change (95% CI) | 14.6 (11.1, 18.0) | 9.4 (5.9, 12.8) |
| LSM difference to placebo (95% CI) | 5.2 (1.8, 8.6) | NA |
| p-value (two-sided) | 0.003 | NA |
| Change from baseline to Week 2 |  |  |
| n | 57 | 55 |
| LSM change (95% CI) | 16.9 (13.1, 20.6) | 8.6 (4.8, 12.4) |
| LSM difference to placebo (95% CI) | 8.3 (4.2, 12.3) | NA |
| p-value (two-sided) | <0.001 | NA |
| Change from baseline to Week 3 |  |  |
| n | 57 | 55 |
| LSM change (95% CI) | 16.8 (12.8, 20.7) | 10.5 (6.5, 14.6) |
| LSM difference to placebo (95% CI) | 6.2 (1.7, 10.7) | NA |
| p-value (two-sided) | 0.007 | NA |
| Change from baseline to Week 4 |  |  |
| n | 55 | 54 |
| LSM change (95% CI) | 16.4 (12.3, 20.5) | 11.1 (6.9, 15.2) |
| LSM difference to placebo (95% CI) | 5.3 (0.6, 10.1) | NA |
| p-value (two-sided) | 0.029 | NA |
| **VAS quality of sleep, mm** |  |  |
| Change from baseline to Week 1 |  |  |
| n | 57 | 55 |
| LSM change (95% CI) | 16.4 (13.2, 19.7) | 10.4 (7.1, 13.7) |
| LSM difference to placebo (95% CI) | 6.0 (2.4, 9.6) | NA |
| p-value (two-sided) | 0.002 | NA |
| Change from baseline to Week 2 |  |  |
| n | 57 | 55 |
| LSM change (95% CI) | 18.0 (14.1, 22.0) | 11.7 (7.6, 15.7) |
| LSM difference to placebo (95% CI) | 6.4 (1.5, 11.2) | NA |
| p-value (two-sided) | 0.011 | NA |
| Change from baseline to Week 3 |  |  |
| n | 57 | 55 |
| LSM change (95% CI) | 18.8 (14.8, 22.7) | 12.2 (8.1, 16.2) |
| LSM difference to placebo (95% CI) | 6.6 (1.7, 11.5) | NA |
| p-value (two-sided) | 0.009 | NA |
| Change from baseline to Week 4 |  |  |
| n | 55 | 54 |
| LSM change (95% CI) | 18.4 (14.0, 22.7) | 13.1 (8.7, 17.5) |
| LSM difference to placebo (95% CI) | 5.3 (-0.2, 10.8) | NA |
| p-value (two-sided) | 0.058 | NA |
| **VAS daytime alertness, mm** |  |  |
| Change from baseline to Week 1 |  |  |
| n | 58 | 56 |
| LSM change (95% CI) | 10.3 (7.1, 13.5) | 7.7 (4.5, 11.0) |
| LSM difference to placebo (95% CI) | 2.5 (-1.1, 6.2) | NA |
| p-value (two-sided) | 0.173 | NA |
| Change from baseline to Week 2 |  |  |
| n | 58 | 56 |
| LSM change (95% CI) | 13.5 (9.7, 17.4) | 9.8 (5.9, 13.8) |
| LSM difference to placebo (95% CI) | 3.7 (-1.1, 8.5) | NA |
| p-value (two-sided) | 0.132 | NA |
| Change from baseline to Week 3 |  |  |
| n | 58 | 56 |
| LSM change (95% CI) | 16.2 (12.5, 19.9) | 10.6 (6.8, 14.4) |
| LSM difference to placebo (95% CI) | 5.6 (1.0, 10.2) | NA |
| p-value (two-sided) | 0.018 | NA |
| Change from baseline to Week 4 |  |  |
| n | 56 | 55 |
| LSM change (95% CI) | 13.9 (9.9, 17.8) | 11.7 (7.7, 15.7) |
| LSM difference to placebo (95% CI) | 2.2 (-2,8, 7.2) | NA |
| p-value (two-sided) | 0.382 | NA |
| **VAS ability to function, mm** |  |  |
| Change from baseline to Week 1 |  |  |
| n | 58 | 56 |
| LSM change (95% CI) | 10.1 (7.0, 13.2) | 7.1 (3.9, 10.2) |
| LSM difference to placebo (95% CI) | 3.0 (-0.6, 6.6) | NA |
| p-value (two-sided) | 0.101 | NA |
| Change from baseline to Week 2 |  |  |
| n | 58 | 56 |
| LSM change (95% CI) | 12.1 (8.3, 15.9) | 9.0 (5.1, 12.9) |
| LSM difference to placebo (95% CI) | 3.1 (-1.7, 8.0) | NA |
| p-value (two-sided) | 0.201 | NA |
| Change from baseline to Week 3 |  |  |
| n | 58 | 56 |
| LSM change (95% CI) | 14.4 (10.8, 18.0) | 9.5 (5.8, 13.1) |
| LSM difference to placebo (95% CI) | 4.9 (0.4, 9.4) | NA |
| p-value (two-sided) | 0.032 | NA |
| Change from baseline to Week 4 |  |  |
| n | 56 | 55 |
| LSM change (95% CI) | 13.0 (9.1, 16.8) | 9.4 (5.5, 13.2) |
| LSM difference to placebo (95% CI) | 3.6 (-1.2, 8.4) | NA |
| p-value (two-sided) | 0.141 | NA |

CI, confidence interval; LSM, least square mean; NA, not applicable; VAS, visual analog score. Linear mixed-effects model for repeated measures: Change from baseline in VAS parameter = treatment group + baseline VAS parameter + week + period + treatment group x week. The VAS scores range from 0 to 100 where a higher score indicates a better outcome.

**Table S4 Proportion of patients with a reduction from baseline to less than two voids per night at Week 1 and 4**

| **Nocturnal voids per night** | **Daridorexant 50 mg** | **Placebo** |
| --- | --- | --- |
| **Week 1** |  |  |
| N | 58 | 58 |
| < 2 | 27 (47%) | 11 (19%) |
| ≥ 2 | 31 (53%) | 47 (81%) |
| **Week 4** | | |
| N | 55 | 57 |
| < 2 | 29 (53%) | 18 (32%) |
| ≥ 2 | 26 (47%) | 39 (68%) |

**Table S5 Change from baseline in IDSIQ total and domain scores at Weeks 1, 2, 3 and 4**

|  | **Daridorexant 50 mg** | **Placebo** |
| --- | --- | --- |
| **IDSIQ total score (0-140)** |  |  |
| Change from baseline to Week 1 |  |  |
| n | 58 | 56 |
| LSM change (95% CI) | -16.6 (-20.8, -12.3) | -11.0 (-15.3, -6.7) |
| LSM difference to placebo (95% CI) | -5.6 (-10.3, -0.9) | NA |
| p-value (two-sided) | 0.021 | NA |
| Change from baseline to Week 2 |  |  |
| n | 58 | 56 |
| LSM change (95% CI) | -20.1 (-25.1, -15.0) | -14.5 (-19.6, -9.4) |
| LSM difference to placebo (95% CI) | -5.6 (-11.7, 0.6) | NA |
| p-value (two-sided) | 0.075 | NA |
| Change from baseline to Week 3 |  |  |
| n | 58 | 56 |
| LSM change (95% CI) | -21.9 (-26.7, -17.0) | -15.9 (-20.8, -11.0) |
| LSM difference to placebo (95% CI) | -6.0 (-11.7, -0.2) | NA |
| p-value (two-sided) | 0.043 | NA |
| Change from baseline to Week 4 |  |  |
| n | 56 | 55 |
| LSM change (95% CI) | -20.9 (-26.2, -15.7) | -15.9 (-21.2, -10.6) |
| LSM difference to placebo (95% CI) | -5.0 (-11.4, 1.4) | NA |
| p-value (two-sided) | 0.122 | NA |
| **IDSIQ sleepiness domain (0-40)** |  |  |
| Change from baseline to Week 1 |  |  |
| n | 58 | 56 |
| LSM change (95% CI) | -5.1 (-6.5, -3.6) | -3.5 (-5.0, -2.1) |
| LSM difference to placebo (95% CI) | -1.5 (-3.1, 0.1) | NA |
| p-value (two-sided) | 0.058 | NA |
| Change from baseline to Week 2 |  |  |
| n | 58 | 56 |
| LSM change (95% CI) | -6.2 (-7.9, -4.6) | -4.7 (-6.4. -3.1) |
| LSM difference to placebo (95% CI) | -1.5 (-3.5, 0.5) | NA |
| p-value (two-sided) | 0.130 | NA |
| Change from baseline to Week 3 |  |  |
| n | 58 | 56 |
| LSM change (95% CI) | -6.6 (-8.2, -5.0) | -5.0 (-6.6, -3.5) |
| LSM difference to placebo (95% CI) | -1.6 (-3.4, 0.3) | NA |
| p-value (two-sided) | 0.090 | NA |
| Change from baseline to Week 4 |  |  |
| n | 56 | 55 |
| LSM change (95% CI) | -6.3 (-8.0, -4.6) | -5.2 (-6.9, -3.5) |
| LSM difference to placebo (95% CI) | -1.1 (-3.2, 0.9) | NA |
| p-value (two-sided) | 0.282 | NA |
| **IDSIQ alert/cognition domain (0-60)** |  |  |
| Change from baseline to Week 1 |  |  |
| n | 58 | 56 |
| LSM change (95% CI) | -6.5 (-8.3, -4.7) | -4.6 (-6.4, -2.7) |
| LSM difference to placebo (95% CI) | -1.9 (-3.9, 0.0) | NA |
| p-value (two-sided) | 0.055 | NA |
| Change from baseline to Week 2 |  |  |
| n | 58 | 56 |
| LSM change (95% CI) | -8.2 (-10.2, -6.1) | -5.7 (-7.8, -3.6) |
| LSM difference to placebo (95% CI) | -2.4 (-4.9, 0.0) | NA |
| p-value (two-sided) | 0.051 | NA |
| Change from baseline to Week 3 |  |  |
| n | 58 | 56 |
| LSM change (95% CI) | -8.9 (-10.9, -6.9) | -6.3 (-8.3, -4.3) |
| LSM difference to placebo (95% CI) | -2.5 (-4.9, -0.2) | NA |
| p-value (two-sided) | 0.032 | NA |
| Change from baseline to Week 4 |  |  |
| n | 56 | 55 |
| LSM change (95% CI) | -8.3 (-10.4, -6.2) | -6.4 (-8.6, -4.3) |
| LSM difference to placebo (95% CI) | -1.9 (-4.4, 0.6) | NA |
| p-value (two-sided) | 0.140 | NA |
| **IDSIQ mood domain (0-40)** |  |  |
| Change from baseline to Week 1 |  |  |
| n | 58 | 56 |
| LSM change (95% CI) | -5.1 (-6.4, -3.7) | -2.9 (-4.2, -1.6) |
| LSM difference to placebo (95% CI) | -2.1 (-3.6, -0.7) | NA |
| p-value (two-sided) | 0.004 | NA |
| Change from baseline to Week 2 |  |  |
| n | 58 | 56 |
| LSM change (95% CI) | -5.7 (-7.3, -4.0) | -4.0 (-5.7, -2.4) |
| LSM difference to placebo (95% CI) | -1.6 (-3.6, 0.4) | NA |
| p-value (two-sided) | 0.109 | NA |
| Change from baseline to Week 3 |  |  |
| n | 58 | 56 |
| LSM change (95% CI) | -6.4 (-8.0, -4.6) | -4.6 (-6.2, -2.9) |
| LSM difference to placebo (95% CI) | -1.8 (-3.8, 0.1) | NA |
| p-value (two-sided) | 0.60) | NA |
| Change from baseline to Week 4 |  |  |
| n | 56 | 55 |
| LSM change (95% CI) | -6.3 (-8.0, -4.6) | -4.3 (-6.0, -2.5) |
| LSM difference to placebo (95% CI) | -2.0 (-4.1, 0.0) | NA |
| p-value (two-sided) | 0.053 | NA |

CI, confidence interval; IDSIQ, Insomnia Daytime symptoms and Impacts Questionnaire; LSM, least square mean; NA, not applicable. Linear mixed-effects model for repeated measures: Change from baseline in IDSIQ parameter = treatment group + baseline IDSIQ parameter + week + period + treatment group x week. Lower IDSIQ scores indicate better outcomes.

**Table S6 ICIQ-NQoL score at baseline and Week 4**

|  | **Daridorexant 50 mg** | **Placebo** |
| --- | --- | --- |
| Baseline |  |  |
| n | 59 | 59 |
| Mean (SD) | 31.7 (7.4) | 31.7 (7.4) |
| Week 4 |  |  |
| N | 58 | 57 |
| Mean (SD) | 17.0 (9.8) | 21.2 (9.6) |

ICIQ-NQoL quality of life relative to nocturia is a score ranging from 0-48 (based on 12 questions of range 0 to 4). Higher scores indicate more nocturia-related issues.

**Table S7 EQ-5D-3L scores at baseline, Week 2 and Week 4**

|  | **Daridorexant 50 mg** | **Placebo** |
| --- | --- | --- |
| Baseline |  |  |
| n | 60 | 60 |
| Mean (SD) | 0.9007 (0.1002) | 0.9007 (0.1002) |
| Week 2 |  |  |
| N | 56 | 56 |
| Mean (SD) | 0.9075 (0.1054) | 0.8929 (0.1188) |
| Week 4 |  |  |
| N | 59 | 58 |
| Mean (SD) | 0.9366 (0.1109) | 0.9245 (0.1003) |

Mean (standard deviation) EQ-5D-3L score (scale 0-1) at baseline, Week 2 and Week 4. Higher scores indicate better quality of life.

**Table S8 Change from baseline in morning sleepiness as assessed by VAS at Week 1, 2, 3 and 4**

|  | **Daridorexant 50 mg** | **Placebo** |
| --- | --- | --- |
| **VAS morning sleepiness, mm** |  |  |
| Change from baseline to Week 1 |  |  |
| n | 57 | 55 |
| LSM change (95% CI) | 11.4 (7.6, 15.1) | 10.7 (7.0, 14.5) |
| LSM difference to placebo (95% CI) | 0.6 (-2.8, 4.1) | NA |
| Change from baseline to Week 2 |  |  |
| n | 57 | 55 |
| LSM change (95% CI) | 13.7 (9.7, 17.8) | 12.6 (8.4, 16.7) |
| LSM difference to placebo (95% CI) | 1.2 (-3.1, 5.5) | NA |
| Change from baseline to Week 3 |  |  |
| n | 57 | 55 |
| LSM change (95% CI) | 16.2 (12.2, 20.3) | 12.6 (8.4, 16.7) |
| LSM difference to placebo (95% CI) | 3.7 (-0.6, 7.9) | NA |
| Change from baseline to Week 4 |  |  |
| n | 55 | 54 |
| LSM change (95% CI) | 15.6 (11.5, 19.7) | 12.2 (8.0, 16.3) |
| LSM difference to placebo (95% CI) | 3.4 (-0.9, 7.8) | NA |

CI, confidence interval; LSM, least square mean; NA, not applicable; VAS, visual analog score. Linear mixed-effects model for repeated measures: Change from baseline in VAS parameter = treatment group + baseline VAS parameter + week + period + treatment group x week. The VAS score ranges from 0 to 100; from 0 ‘very sleepy’ to 100 ‘not sleepy at all’. A higher score indicates less morning sleepiness.

**Figure S1 Time from bedtime to first nocturnal void at Week 1 and 4**

**
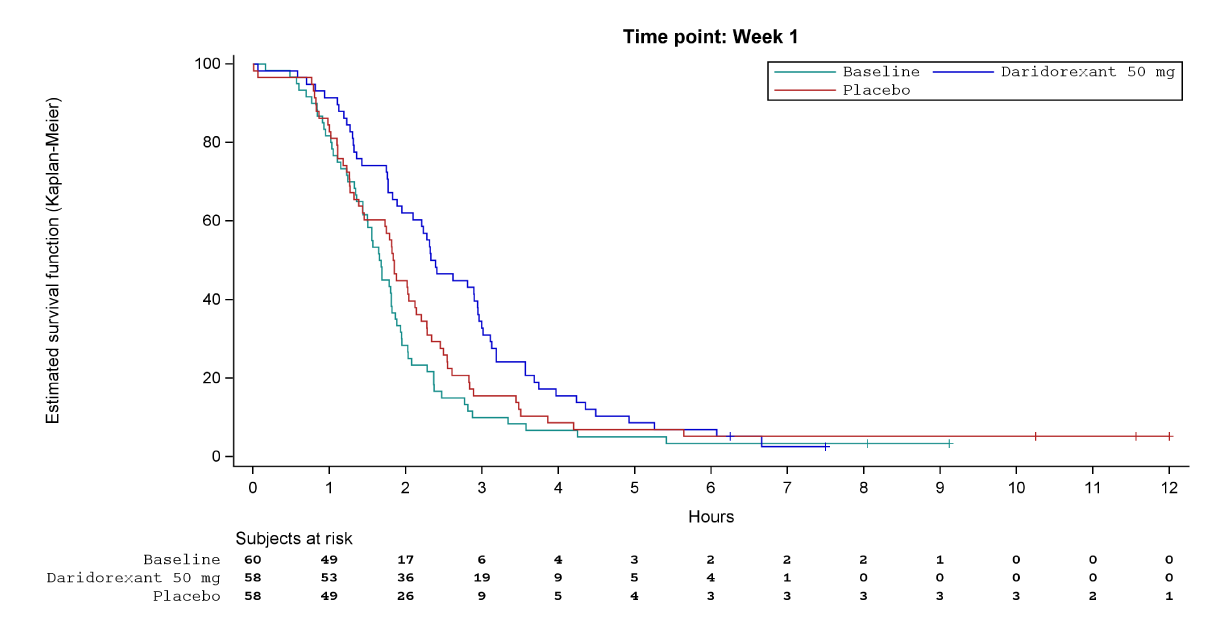
**

**
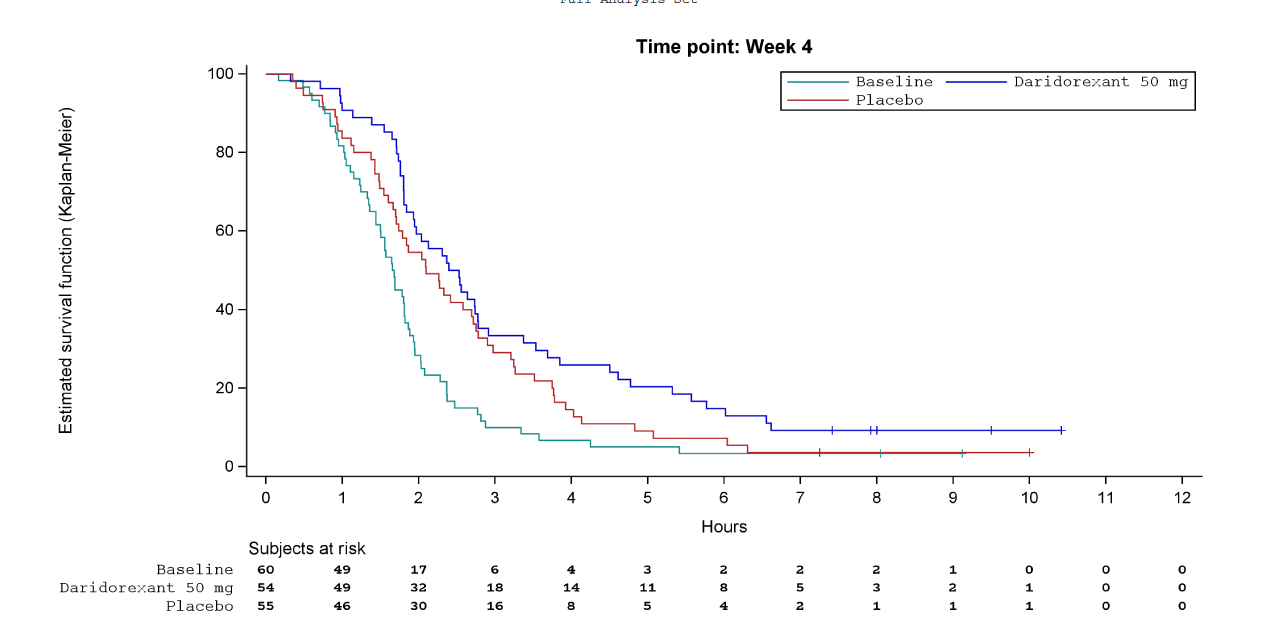
**

Time to first nocturnal void (in hours) = time of first nocturnal void (after bedtime and before waketime) – bedtime. The average time to first void (with a volume >0 ml) computed across the three voiding nights (by visit). Baseline is displayed as a reference. The same patients contribute to all three KM-curves, with differences in sample size caused by drop-out or missing data.

**List of investigators and sites**

| **Principal Investigator** | **Country** | **City** | **Institution Type** | **Site Name** |
| --- | --- | --- | --- | --- |
| [Heike Benes](https://idorsia-clinical.veevavault.com/ui/#object/study_person__clin/OOZ0000000I0169) | Germany | Schwerin | Clinical Research Unit | SOMNIBENE Institut für Medizinische Forschung und Schlafmedizin Schwerin GmbH |
| [Jörg Horstmann](https://idorsia-clinical.veevavault.com/ui/#object/study_person__clin/OOZ0000000I7005) | Germany | Aachen | Hospital | Praxisklinik am Franziskushospital - Urologisches Zentrum Euregio |
| [Katharina Lederer](https://idorsia-clinical.veevavault.com/ui/#object/study_person__clin/OOZ0000000I8008) | Germany | Berlin | Clinical Research Unit | ASR Advanced Sleep Research GmBH |
| [Eva Hellmis](https://idorsia-clinical.veevavault.com/ui/#object/study_person__clin/OOZ0000000IC019) | Germany | Duisburg | Physician’s Office | Urologicum Duisburg |
| [Michael Berse](https://idorsia-clinical.veevavault.com/ui/#object/study_person__clin/OOZ0000000IE001) | Germany | Duisburg | Physician’s Office | Gemeinschaftspraxis Michael Berse & Hans Schippel |
| [David Castro Díaz](https://idorsia-clinical.veevavault.com/ui/#object/study_person__clin/OOZ0000000HW157) | Spain | San Cristóbal de La Laguna | Hospital | Hospitalario Universitario de Canarias |
| [Jose Emilio Batista Miranda](https://idorsia-clinical.veevavault.com/ui/#object/study_person__clin/OOZ0000000HT162) | Spain | Barcelona | Physician’s Office | UROCLINICA Barcelona |
| [Jose Maria Sanchez Merino](https://idorsia-clinical.veevavault.com/ui/#object/study_person__clin/OOZ0000000HV173) | Spain | A Coruña | Hospital | Complejo Hospitalario Universitario A Coruña |
| [Mateo Hevia Suarez](https://idorsia-clinical.veevavault.com/ui/#object/study_person__clin/OOZ0000000KT056) | Spain | Oviedo | Hospital | Hospital Universitario Central de Asturias (HUCA) |
| [Jose Luis Alvarez-Ossorio Fernandez](https://idorsia-clinical.veevavault.com/ui/#object/study_person__clin/OOZ0000000I0027) | Spain | Cadiz | Hospital | Hospital Universitario Puerta del Mar (HUPM) |
| [Hector Garde García](https://idorsia-clinical.veevavault.com/ui/#object/study_person__clin/OOZ0000000HZ079) | Spain | Madrid | Hospital | Hospital Universitario Fundación Jiménez Díaz |
| [Jose Miguel Gomez de Vicente](https://idorsia-clinical.veevavault.com/ui/#object/study_person__clin/OOZ0000000HX121) | Spain | Madrid | Hospital | Hospital Universitario La Paz |
| [Alfredo Rodriguez Rodriguez](https://idorsia-clinical.veevavault.com/ui/#object/study_person__clin/OOZ0000000IE004) | Spain | Barcelona | Hospital | Parc de Salut Mar / Hospital del Mar |
| [Ashley Baker](https://idorsia-clinical.veevavault.com/ui/#object/study_person__clin/OOZ0000000KU148) | United States | Shreveport | Clinical Research Unit | Ochsner LSU Health Shreveport-Regional Urology |
| [Franklin Chu](https://idorsia-clinical.veevavault.com/ui/#object/study_person__clin/OOZ0000000L4040) | United States | San Bernardino | Clinical Research Unit | San Bernardino Urological Associates Medical Group |
| [Fernando Bianco](https://idorsia-clinical.veevavault.com/ui/#object/study_person__clin/OOZ0000000OZ030) | United States | Hialeah | Physician’s Office | Urology Research Network |
| [Sylvia Shoffner](https://idorsia-clinical.veevavault.com/ui/#object/study_person__clin/OOZ0000000JT095) | United States | Raleigh | Clinical Research Unit | Accellacare Research of Cary |
| [Alan Fine](https://idorsia-clinical.veevavault.com/ui/#object/study_person__clin/OOZ0000000PG002) | United States | Pompano Beach | Clinical Research Unit | CLINICAL RESEARCH CENTER OF FLORIDA |
| [Gene Neytman](https://idorsia-clinical.veevavault.com/ui/#object/study_person__clin/OOZ0000000TS051) | United States | Miami Beach | Clinical Research Unit | Quantum Clinical Trials |
| [Sandro Bacchelli](https://idorsia-clinical.veevavault.com/ui/#object/study_person__clin/OOZ0000000UO006) | United States | Weston | Clinical Research Unit | Encore Medical Research of Weston |
| [Ramon Berenguer](https://idorsia-clinical.veevavault.com/ui/#object/study_person__clin/OOZ0000000UF039) | United States | Miramar | Clinical Research Unit | Innovia Research Center Inc |

**References**

1. Phillips-Beyer, A., Kawata, A. K., Kleinman, L., Seboek Kinter, D. & Flamion, B. (2024) Meaningful Within-Patient Change in Subjective Total Sleep Time in Patients with Insomnia Disorder: An Analysis of the Sleep Diary Questionnaire Using Data from Open-Label and Phase III Clinical Trials, *Pharmaceut Med.* **38**, 133-144.

2. Morin, C. M., Belleville, G., Bélanger, L. & Ivers, H. (2011) The Insomnia Severity Index: psychometric indicators to detect insomnia cases and evaluate treatment response, *Sleep.* **34**, 601-8.

3. Hudgens, S., Phillips-Beyer, A., Newton, L., Seboek Kinter, D. & Benes, H. (2021) Development and validation of the Insomnia Daytime Symptoms and Impacts Questionnaire (IDSIQ), *Patient.* **14**, 249-268.

4. Phillips-Beyer, A., Kawata, A. K., Kleinman, L. & Kinter, D. S. (2023) Meaningful within-patient change on the Insomnia Daytime Symptoms and Impacts Questionnaire (IDSIQ): Analysis of phase III clinical trial data of daridorexant, *Pharmaceutical Medicine.* **37**, 291-303.

5. Phillips-Beyer, A., Olivieri, A., Marco, T. D. & Kimel, M. (2023) 0384 Assessing the content validity of the IDSIQ through cognitive interviews in individuals with comorbid insomnia and nocturia, *Sleep.* **46**, A170-A171.

6. Bright, E., Cotterill, N., Drake, M. & Abrams, P. (2014) Developing and validating the International Consultation on Incontinence Questionnaire bladder diary, *Eur Urol.* **66**, 294-300.

7. Rabin, R. & de Charro, F. (2001) EQ-5D: a measure of health status from the EuroQol Group, *Ann Med.* **33**, 337-43.

8. Devlin, N. J. & Brooks, R. (2017) EQ-5D and the EuroQol Group: Past, Present and Future, *Appl Health Econ Health Policy.* **15**, 127-137.

9. Abraham, L., Hareendran, A., Mills, I. W., Martin, M. L., Abrams, P., Drake, M. J., MacDonagh, R. P. & Noble, J. G. (2004) Development and validation of a quality-of-life measure for men with nocturia, *Urology.* **63**, 481-6.
